# Evaluation of Changes in Central Macular Thickness After Neodymium-Doped Yttrium Aluminum Garnet (Nd:YAG) Posterior Capsulotomy: An Optical Coherence Tomography-Based Prospective Observational Study

**DOI:** 10.7759/cureus.104377

**Published:** 2026-02-27

**Authors:** Himanshu Singh, Rituka Gupta, Deepanshi Agrawal, Khushali Solanki, Anagha Chauhan, Khalid Khan, Priyanka

**Affiliations:** 1 Ophthalmology, Government Medical College Singrauli, Singrauli, IND; 2 Ophthalmology, Lakshmi Narain College of Technology, Indore, IND; 3 Ophthalmology, Chirayu Medical College and Hospital, Bhopal, IND; 4 Community Medicine, Government Medical College Singrauli, Singrauli, IND; 5 Ophthalmology, All India Institute of Medical Sciences, Bhopal, Bhopal, IND

**Keywords:** best-corrected visual acuity (bcva), central macular thickness, nd:yag laser, optical coherence tomography, posterior capsule opacification

## Abstract

Background

After cataract surgery, one of the most common complications causing diminution of vision is posterior capsular opacification (PCO). Neodymium-doped yttrium aluminum garnet (Nd:YAG) laser therapy is currently the most popular option for treating PCO, as it is a fast, non-invasive, and successful procedure.

Methodology

This prospective observational study was conducted at a tertiary eye care center in central India. A total of 64 pseudophakic patients were included. Preoperative evaluation included a detailed demographic history and complete ophthalmological examination, including visual acuity, intraocular pressure (IOP), and central macular thickness (CMT). All patients underwent Nd:YAG laser capsulotomy for PCO. Following laser treatment, IOP, best-corrected visual acuity (BCVA), and CMT were evaluated after three and eight weeks. Optical coherence tomography (OCT) was used to measure CMT. The data analysis was done using suitable statistical tests.

Results

A total of 64 patients who met the inclusion criteria were enrolled. There was a significant and stable improvement in BCVA, with mean BCVA improving from baseline 0.82 ± 0.15 logMAR preoperatively to 0.12 ± 0.09 logMAR postoperatively (p < 0.001). Regarding CMT values, post-laser macular thickness at three weeks (mean ± SD = 241.50 ± 10.76) was significantly increased (p < 0.001) compared to pre-laser macular thickness (mean ± SD = 222.89 ± 9.23); however, it was statistically non-significant at eight weeks (p = 0.08). Most patients remained within the 10-16 mmHg IOP range throughout the follow-up period.

Conclusions

Nd:YAG laser posterior capsulotomy is a quick, safe, and non-invasive procedure for PCO. Increased macular thickness was reported as a complication after performing YAG laser posterior capsulotomy, lasting for a substantial period, which was revealed on follow-up with OCT. As this was not clinically observed, it did not necessitate regular prophylactic treatment.

## Introduction

Posterior capsular opacification (PCO) is one of the most common complications following uncomplicated cataract surgery. It is caused by the proliferation, migration, and differentiation of lens epithelial cells following cataract extraction [1]. During the first five postoperative years, the incidence of PCO is seen in 3% to 50% of patients undergoing uncomplicated cataract surgery.

Pseudophakic patients with PCO mainly complain of a decline in visual acuity and altered contrast sensitivity [2,3]. Neodymium-doped yttrium aluminum garnet (Nd:YAG) posterior capsulotomy is a quick, non-invasive, outpatient, safe procedure [4]. However, some complications have been documented, such as iritis, intraocular lens (IOL) pitting, uveitis, vitreous prolapse, cystoid macular edema (CME), IOP elevation, and retinal detachment [5-7]. According to Holladay et al., the size of optimal capsulotomy should be equal to or greater than the scotopic pupil size within the border of the IOL [8].

Although IOP elevation is usually transient, it is the most prevalent complication of YAG laser posterior capsulotomy. It results either due to release of capsular debris, vitreous debris, cells in the anterior chamber after capsulotomy blocking the trabecular meshwork, an increase in the ciliary epithelium secretion by the shockwaves produced by the YAG laser, or inflammatory edema of the ciliary body resulting in a pupillary block [6,9]. Macular edema following YAG laser capsulotomy is caused due to damage of the blood-aqueous barrier leading to the release of inflammatory mediators and increasing the permeability of perifoveal capillaries [10]. Different studies have reported the incidence of CME in 0% to 4.3% of cases after Nd:YAG posterior capsulotomy [11].

According to previous studies, the duration and severity of raised IOP and macular edema are found to be lower when less than 80 mJ of total energy is used [12]. Various commercially available imaging tools are available to measure macular thickness, including optical coherence tomography (OCT), retinal thickness analyzer, and confocal scanning laser ophthalmoscope. Among these, OCT is the most commonly employed imaging tool for macular assessment, offering direct visualization of the different layers of the retina [13].

Energy from Nd:YAG laser capsulotomy can damage retinal tissue due to thermal photocoagulation and can influence the central part of the retina by changing macular thickness. The current study aimed to evaluate the impact of YAG laser capsulotomy on the central macular thickness (CMT) using OCT in central India.

## Materials and methods

This single-center, hospital-based, prospective, observational study was conducted between March 2023 and December 2024 at a tertiary eye care center in central India. All patients diagnosed with PCO following uneventful cataract surgery with posterior chamber IOL implantation visiting the outpatient Department of Ophthalmology, Chirayu Medical College and Hospital were enrolled in the study. The study was approved by the Institutional Ethics Committee at Chirayu Medical College and Hospital, Bhopal (approval number: CMCH/IEC/2022/59). The study was conducted in accordance with the tenets of the Declaration of Helsinki and its later amendments. Informed consent was obtained from all patients. Patients with complicated cataract surgery, uveitis, glaucoma, trauma, retinopathy, maculopathy, or any media opacity interfering with OCT evaluation were excluded from the study.

A detailed demographic history, including age, gender, residence, and occupation, was obtained, and a complete ocular examination was performed for every patient. Ophthalmic evaluation included best-corrected visual acuity (BCVA) by Snellen’s chart and anterior-segment examination by slit-lamp biomicroscopy. Fundus evaluation was performed using a 90 D lens with slit lamp or indirect ophthalmoscopy after pupillary dilatation with eye drop tropicamide 0.8% and phenylephrine 5%. Baseline IOP was measured by the Goldmann applanation tonometer. For each patient, baseline CMT was determined preoperatively using spectral-domain OCT. All OCT imaging was performed by a single technician using a single OCT machine (PRIMUS 200, Carl Zeiss Meditec AG, Jena, Germany). The procedure was clearly explained to the patient, and they were asked to maintain steady fixation during the procedure. Topical anesthesia was achieved using eye drop proparacaine 0.5%. During the capsulotomy, a contact laser capsulotomy lens with a lubricating gel was used. The Nd:YAG laser posterior capsulotomy was performed using a Q-switched Nd:YAG laser device (VISULAS YAG III, Carl Zeiss Meditec AG, Jena, Germany) after pupillary dilation with eye drop tropicamide in a single session. After capsulotomy, all patients were prescribed eye drops prednisolone acetate 1% four times a day and timolol maleate 0.5% two times daily for a period of five days. Post-procedural visual acuity, IOP, and CMT measurements were noted at three and eight weeks. The total laser energy and the number of shots utilized were recorded, and any complications were documented.

Statistical analysis

The data were serially entered in a Microsoft Excel (Microsoft Corp., Redmond, WA, USA) worksheet and analyzed using SPSS version 25 (IBM Corp., Armonk, NY, USA). The mean and standard deviation (SD) were used to present all the descriptive and demographic data. The qualitative variables were displayed as numbers and percentages. A paired t-test was applied to test the significant difference in BCVA over time. The significant difference in CMT over time (preoperatively and three and eight weeks postoperatively) was calculated using the Friedman test and the Wilcoxon signed-rank test. Further, multiple linear regression was applied to determine the significant predictors for the change in CMT over time. Only a two-tailed p-value <0.05 was considered statistically significant, and all confidence intervals (CIs) described were true for 95% of the sample.

## Results

A total of 64 patients who met the inclusion criteria were enrolled in the analysis. The age of the patients ranged from 20 to 87 years, with a mean age of 64.1 ± 13.2 years. Overall, 26 (40.6%) were males, and 38 (59.4%) were females. The right eye (37, 57.8%) involvement was higher compared to the left eye (27, 42.2%). Phacoemulsification was performed in 34 (53.1%) and small-incision cataract surgery in 30 (46.9%) cases (Table 1).

**Table 1:** Demographic profile.

Characteristics		Frequency (percentage)
Age (years)	Mean ± SD	64.1 ± 13.2 years
	20–40	4 (6.2%)
	40–60	16 (25%)
	>60	44 (68.8%)
Sex	Male	26 (40.6%)
	Female	38 (59.4%)
Laterality	Right eye	37 (57.8%)
	Left eye	27 (42.2%)
Type of surgery	Phacoemulsification	34 (53.1%)
	Small-incision cataract surgery	30 (46.9%)

The duration between cataract surgery and laser capsulotomy varied from 1 to 7.5 years, with a mean of 4.5 ± 1.75 years. The mean total shot count was 19.0 ± 4.6 with a range of 12 to 36 shots. The mean energy level used was 32.3 ± 7.9 mJ, ranging from 18 to 54 mJ (Table 2). Most patients remained within the 10-16 mmHg IOP range throughout the follow-up period. No statistically significant changes in IOP were found after laser treatment.

**Table 2:** Baseline characteristics. CMT = central macular thickness

Parameter	Mean ± SD	Minimum–maximum
Age (years)	64.1 ± 13.2	20–87
Duration (years)	4.5 ± 1.75	1–7.5
Pre-laser CMT (µm)	222.89 ± 9.23	190–240
Laser energy (mJ)	32.3 ± 7.9	18–54
Number of shots	19.0 ± 4.6	12–36
Post-laser CMT (3 weeks) (µm)	241.50 ± 10.76	208–265
Post-laser CMT (8 weeks) (µm)	235.08 ± 9.86	204–256

The mean preoperative BCVA measurement was 0.82 ± 0.15 logMAR, which showed significant improvement to 0.13 ± 0.12 logMAR by three weeks and further to 0.12 ± 0.09 logMAR at eight weeks postoperatively, indicating substantial visual recovery (Table 3).

**Table 3:** Change in BCVA pre-laser and post-laser treatment using ANOVA. BCVA = best-corrected visual acuity; ANOVA = analysis of variance

BCVA	Mean ± SD	P-value
Pre-laser BCVA (logMAR)	0.82 ± 0.15	<0.001
Post-laser BCVA (logMAR) at 3 weeks	0.13 ± 0.12	
Post-laser BCVA (logMAR) at 8 weeks	0.12 ± 0.09	

Paired t-test analysis showed a significant improvement in BCVA after laser treatment. Mean logMAR improved from baseline to three weeks by 0.69 (95% CI = 0.65-0.74; p < 0.001) and to eight weeks by 0.71 (95% CI = 0.67-0.75; p < 0.001). Further, a minor but statistically significant improvement was noted between three and eight weeks (mean difference = 0.017; p = 0.004), indicating stabilization of visual acuity (Table 4).

**Table 4:** Pairwise comparison of the change in BCVA using the paired t-test. BCVA = best-corrected visual acuity

	Paired differences					P-value
	Mean	Standard deviation	Standard error mean	95% confidence interval of the difference		
				Lower	Upper	
LogMAR (pre-laser) – logMAR (3 weeks)	0.69245	0.17194	0.02149	0.64950	0.73540	0.000
LogMAR (pre-laser) – logMAR (8 weeks)	0.70966	0.16461	0.02058	0.66854	0.75078	0.000
LogMAR (3 weeks) – logMAR (8 weeks)	0.01722	0.04658	0.00582	0.00558	0.02885	0.004

Regarding CMT, the preoperative CMT ranged from 190 to 240 µm (mean ± SD = 222.89 ± 9.23). At three weeks, postoperative CMT ranged from 208 to 265 µm (mean ± SD = 241.50 ± 10.76). At eight weeks, postoperative CMT ranged from 204 to 256 µm (mean ± SD = 235.08 ± 9.86).

After checking the normality of the data, the data were not normally distributed; therefore, the Friedman test was applied to check statistical significance. There was a statistically significant difference across pre-and post-laser CMT at three weeks (p < 0.001), while statistically non-significant but clinically significant improvement of CMT at eight weeks (p = 0.08) (Table 5).

**Table 5:** Change in CMT pre-laser and post-laser treatment using the Friedman test. CMT = central macular thickness

CMT (µm)	Mean	Standard deviation	P-value
Pre-laser CMT	222.89	9.23	<0.001
Post-laser CMT (3 weeks)	241.50	10.76	<0.001
Post-laser CMT (8 weeks)	235.08	9.866	0.08

On applying the Wilcoxon signed-rank test for pairwise comparison, there was a statistically significant change in CMT across the time points except between three and eight weeks (Table 6).

**Table 6:** Pairwise comparison of the change in CMT using Wilcoxon signed-rank test. CMT = central macular thickness

	Pre-laser CMT – post-laser CMT (3 weeks)	Pre-laser CMT – post-laser CMT (8 weeks)	Post-laser CMT (8 weeks) – post-laser CMT (3 weeks)
Z value	-6.957	-6.834	-6.973
P-value	<0.001	<0.001	0.082

**Figure 1:**
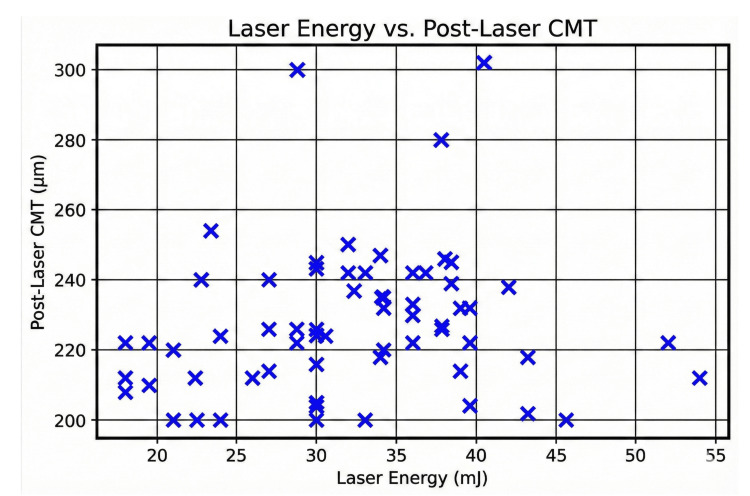
Scatter plot: laser energy versus post-laser CMT. CMT = central macular thickness

We analyzed the relationship between laser energy and CMT after Nd:YAG capsulotomy. While laser energy varied widely among patients, there was a moderate positive correlation between laser energy levels and post-laser CMT, showing that a higher laser energy may be associated with increased CMT (Figure 1).

A multiple linear regression was applied to predict three-week and eight-week post-laser CMT using pre-laser CMT, age, gender, duration between cataract surgery and laser, number of shots, and laser energy as predictors. The model explained 38% of the variance in post-laser CMT at three weeks (R² = 0.38) and was statistically significant (F (7,95) = 4.7, p < 0.001). Similarly, for post-laser CMT at eight weeks (R² = 0.404), the model explained 40% of the variance and was statistically significant (F (5.4,96), p < 0.001). Pre-laser CMT (β = 0.624, p < 0.001 at three weeks post-laser and β = 0.823, p < 0.001 at eight weeks post-laser), age (β = -0.576, p = 0.014 at three weeks post-laser and β = -0.676, p = 0.003 at eight weeks post-laser), and laser energy used (β = 0.106, p = 0.044 at three weeks post-laser and β = 0.174, p = 0.038 at eight weeks post-laser) were significant predictors (Table 7).

**Table 7:** Multiple linear regression analysis between study variables and post-laser CMT at three and eight weeks. CMT = central macular thickness

Variables	Beta coefficient (β) of change in CMT at 3 weeks post-laser	P-value	Beta coefficient (β) of change in CMT at 8 weeks post-laser	P-value
Pre-laser CMT (µm)	0.624	<0.001	0.823	<0.001
Age (years)	-0.576	0.014	-0.676	0.003
Gender	-0.040	0.681	-0.046	0.684
Duration between cataract surgery and laser (years)	-0.134	0.424	-0.098	0.546
Number of shots	0.104	0.571	0.049	0.337
Laser energy (mJ)	0.106	0.044	0.174	0.038

No patient developed hyphema, severe anterior chamber reaction, iritis, vitritis, vitreous prolapse, or retinal detachment during the study. IOL pitting was noted in two patients.

## Discussion

The most common delayed complication after cataract surgery is PCO. The incidence of PCO was found to be 20.7% at two years, rising to 28.5% at five years after uneventful cataract surgery [14]. The most frequent complication after posterior capsulotomy is a transient rise in IOP, which may occur in 15% to 36% of patients not receiving any prophylactic treatment [15]. After the capsulotomy, IOP typically begins to rise immediately, peaking at three to four hours, which may remain elevated for 24 hours before returning to baseline by one week. However, Singh et al. did not find any significant rise in IOP in the groups receiving ocular hypotensive drugs during the same period [15]. Shetty et al. observed a significant change in IOP in the group receiving more than 30 shots [16]. However, Shani et al. and Ari et al. did not find any rise in IOP after Nd:YAG laser capsulotomy [12,17]. Our study had similar findings that could be due to the use of antiglaucoma medication in the postoperative period.

This study observed a significant and stable improvement in BCVA, with mean BCVA improving preoperatively from 0.82 ± 0.15 logMAR to 0.12 ± 0.09 logMAR postoperatively (p < 0.001). This finding was consistent with previous studies and confirms the effectiveness of the procedure in restoring visual function. Aslam et al. also noted significant improvements in BCVA after Nd:YAG capsulotomy with a mean change of 0.3 to 0.4 logMAR units [18]. Patel et al. also found mean BCVA improvement from 0.62 logMAR to 0.22 logMAR postoperatively, similar to our findings [19]. Soujanya et al. observed BCVA improvement after four weeks, reinforcing the long-term benefits of capsulotomy [20]. The correlation of BCVA improvement with the severity of PCO preoperatively has been found, suggesting that early intervention leads to better visual recovery.

In this study, we observed a significant increase in CMT at three weeks compared to the baseline preoperative levels, even with prophylaxis with steroids. The mean CMT increased till three weeks and then started decreasing toward the pre-laser CMT value till eight weeks post-laser. Karahan et al. also reported a significant increase in CMT after one week of Nd:YAG capsulotomy, which returned to baseline levels within one month [21]. However, Ruiz et al. noted little change in macular thickness at three months postoperatively [22]. The similarity of this study with the majority of previous studies is likely due to similar follow-up periods. However, the variation in the results may be due to the differences in patient demographics, the amount of energy applied, and the time elapsed since the initial cataract surgery. Factors other than laser energy, such as baseline CMT, ocular comorbidities, and individual healing responses, may also play a more significant role in determining post-laser macular thickness. The mechanical force resulting from the movement of the vitreous and due to the damage to the blood-aqueous barrier, leading to the release of inflammatory mediators following Nd:YAG capsulotomy, may increase CMT [23].

This study analyzed the relationship between laser energy and CMT after Nd:YAG capsulotomy. The findings suggest that while laser energy varied widely among patients, there was a moderate positive correlation between laser energy levels and post-laser CMT. The scatter plot demonstrated a broad distribution of post-laser CMT values across different laser energy levels. This finding was consistent with Ari et al. [12], who also observed increased macular thickness in patients receiving high-energy treatment compared to the low-energy group, while another study by Steinert et al., on a series of 897 Nd:YAG laser posterior capsulotomies, did not find any relation between the number of laser pulses and energy delivered with macular edema [6]. Similarly, Kara et al. did not find any significant correlation between postoperative macular thickness and the amount of energy applied in their study [24]. Selim et al. did not observe a substantial change in CMT during the first month and did not find any significant change in CMT in relation to the energy levels during the short follow-up period [10]. Aslam et al. found that clinically significant macular edema was not common in those without predisposing risk factors [18]. Buehl et al. found increased macular thickness in some patients due to an inflammatory response [2]. Diabetes, uveitis, or prior retinal disease have been identified as potential risk factors for increased CMT following laser procedures. As our study did not include any patients with the above risk factors, other causes need to be evaluated further, which may lead to susceptibility to post-laser macular edema.

On multiple linear regression analysis, we found age, pre-laser CMT, and laser energy to be significant predictors for post-laser increased macular thickness. However, Altiparmak et al. did not find any significant relationship between macular thickness and variables such as age, gender, number of shots, and energy applied during the treatment [25].

This study has some limitations. Given the smaller sample size and a single-center design with a short follow-up period, the findings cannot be generalized to a larger population. The study did not include patients with ocular disease. Thus, these results could not be applicable to patients with glaucoma who may develop a serious elevation of the IOP after capsulotomy or an increase in CMT in diabetic patients. As this study had a short follow-up period, late complications of capsulotomy, such as posterior vitreous detachment, retinal tear, and retinal detachment, were not evaluated. Further, multicenter studies with a large number of patients and longer follow-up are needed to confirm these findings.

## Conclusions

YAG laser capsulotomy is a non-invasive, outpatient, quick, and relatively safe technique for the management of PCO. This study confirms that Nd:YAG laser posterior capsulotomy significantly improves BCVA in patients with PCO. Increased CMT was reported as a complication after performing YAG laser posterior capsulotomy, which was sustained over a substantial follow-up period with OCT, which may need intervention. As this was not observed clinically, regular prophylactic treatment was not deemed necessary.
